# How Do the Young Perceive Urban Parks? A Study on Young Adults’ Landscape Preferences and Health Benefits in Urban Parks Based on the Landscape Perception Model

**DOI:** 10.3390/ijerph192214736

**Published:** 2022-11-09

**Authors:** Daixin Dai, Mingyang Bo, Youmei Zhou

**Affiliations:** Department of Landscape Architecture, College of Architecture and Urban Planning, Tongji University, Shanghai 200092, China

**Keywords:** youth, landscape perception model, landscape preference, health benefits, Shanghai

## Abstract

(1) Background: Youth’s physical and mental health is of increasing concern today. However, gaining a more comprehensive knowledge of young people’s landscape preferences for urban parks is challenging. Additionally, young adults’ voices (aged from 20 to 24) are often neglected. (2) Methods: This study collected 349 interview questionnaires from 2014 to 2020 and recorded them into Nvivo10. Firstly, the study did a thematic analysis using the preliminary coding framework based on the landscape perception model to code the interview data and statistics on the frequencies of each theme and code. Then, we used diffractive analysis to interpret original materials to comprehend the underlying significance. (3) Results: Our research showed that young adults’ landscape perceptions are richer in diversity and express more subjective feelings. Their landscape preferences are also related to behavioral activities in addition to environmental features, which have some differences from teenagers. (4) Conclusions: It is helpful to attract more young adults by creating sound and smell landscapes, accommodating more dynamic sports and recreation facilities, and controlling unhygienic and noise problems, which can offer better design, planning, and management for creating inclusive urban parks. The landscape perception model developed in this paper can also provide a reference for related studies in the future.

## 1. Introduction

Youth, defined by the UN General Assembly as those between the ages of 15 and 24 (inclusive), are often the most vulnerable group in society, whose physical and mental health is of increasing concern today. It has been confirmed that youth’s contact and interaction with nature positively impact their physical and mental health [1,2], and living in a more natural, green environment could improve people’s physical and mental health. Therefore, urban parks are everyday places for nature contact and social interactions, while the youth do not use parks well [3]. Some studies have shown that youth’s visitation of parks and their level of physical activity while visiting parks are both low [4]. For example, an Australian observational study found that youth only constituted 7% of park visitors [5]. As a result, it is a significant challenge to increase youth’s visitation to parks and improve their nature contact and social interactions for youth health promotion. This paper reports on an in situ qualitative investigation into how the youth perceive urban parks and which health benefits and wellbeing are spontaneously identified by the youth users of urban parks.

The research proposed that urban park is one of the important facilities of the community life circle [6]. The various environmental, aesthetic, and recreational benefits that urban parks provide for citizens are linked to users’ health [7]. Such benefits can be obtained through frequent visits to urban parks, and positive perceptions and experiences during the visit lead to health promotion [8,9]. Furthermore, it has been found that youth’s perceptions in urban parks are more abundant and sensitive, as their experiences in urban parks result in senses of self, escape, connection, and care [10], all of which can help produce many kinds of mental health and wellbeing benefits for youth. Therefore, it is significant to understand youth’s perceived preferences for urban parks, enhance their visitation and use of urban parks, and improve their perceptions and experiences, which can contribute to their physical and mental health.

### 1.1. Previous Research on Youth and Park Perception

Although both are within the age of youth, it is necessary to distinguish between teenagers (aged 13–19) and young adults (aged 20–24), as they are likely to face different social, psychological, and health problems [11]. Public concerns have been shown about teenagers’ insufficient physical activities and mental health crises [12,13]. As a result, research has long discussed how urban parks influence teenagers’ physical and mental health benefits [1,2,3,14]. However, the health issues faced by young adults are being ignored, unlike those that plague teenagers. It is reported that the majority of mental health crises and disorders among young adults result from their early recognition [13], as their mental health and wellbeing are affected by negative pressures such as learning, poverty, electronic products, and social media. In particular, today’s young adults have become the generation most reliant on electronics for communication and connection, significantly impacting their personality and peer pressure [15]. Therefore, we should pay more attention to young adults’ physical and mental health problems to make urban parks bring more health benefits to them.

The importance of natural attributes and environmental features for landscape perception has long been discussed in many studies. Several environmental elements, such as greenery, water bodies, and recreational facilities, are closely related to the perception of urban parks [16,17]. To make parks more attractive to young people, research has identified environmental characteristics that attract them to urban parks [3]. As a result, young people’s perception of urban parks has gained widespread attention. Chinese researchers have developed a regression model of environmental characteristics of urban parks and young people’s restorative perceptions and mapped restorative perceptions to summarize the environmental characteristics that trigger restorative perceptions among young people [18]. Studies have developed a QUality INdex of Parks for Youth (QUINPY) including 18 variables grouped into five key themes: structured play diversity, nature, park size, maintenance, and safety, which measures the quality of urban parks for youth with geospatial data [19]. Much of the existing research focuses on environmental features and physical attributes that influence young people’s park perceptions, which implicitly imply landscape preferences in the objectivist paradigm. However, landscape preferences are not only influenced by environmental features but also related to the way people perceive these environments and their subjective feelings [20]. For example, current research explored the characteristics of the public’s perception of park environments and focused on their positive and negative perceptions of urban parks, to some extent deepening the understanding of the public perception of urban parks [21]. Therefore, it is challenging to gain a more comprehensive knowledge of young people’s landscape preferences for urban parks, as it requires not only finding out the physical attributes that influence their perceptions but also learning their subjective perceptions of urban parks.

### 1.2. Spatial Perception and Landscape Perception

Spatial perception is a real, direct, multiple, and integrated human perceptual experience of the environment. Considered the basis for the establishment of urban and architectural design, spatial perception theory has been applied to the evaluation and quantification of urban public space perceptions [22,23,24]. However, it is challenging to comprehensively analyze people’s perceptual experience of urban parks with spatial perception due to their inherently intangible, subjective, and ambiguous attributes [21]. Furthermore, it is most likely because natural landscapes dominate urban parks, making them unique among urban public spaces. Therefore, it is required to conduct research from the perspective of landscape perception.

Furthermore, the theory of landscape perception suggests that the process of perception is cyclical and interactive rather than unidirectional [25]. Such an idea differs from spatial perception reflecting the transformation of the objective physical environment through the human perceptual system into emotions, attitudes, and behaviors with subjective perceptual properties [26]. Therefore, landscape perception theory can help understand the overall characteristics of young adults’ perception in urban parks because human perceptual experience is a systemic whole in its theoretical framework.

### 1.3. Development of Landscape Perception Model

**Figure 1:**
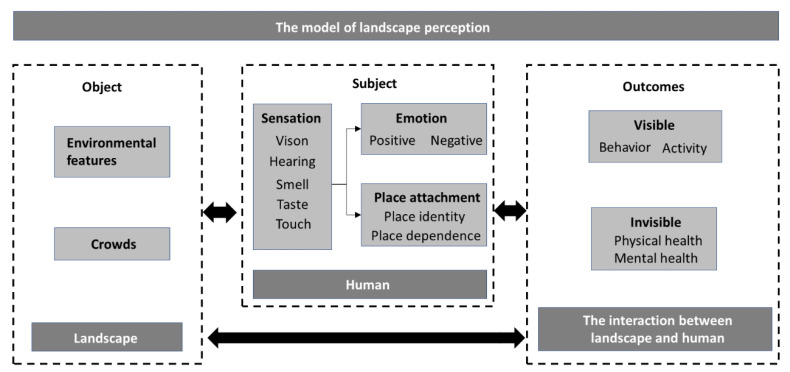
Landscape perception model.

The current research has long discussed the public perception of parks [27]. Other than the perception of environmental features [16,17], positive and negative perceptions have also been taken into consideration in recent research on urban park perceptions [21]. To make the study more reliable, researchers used a health model based on the person-environment-health relationship in the qualitative study of park users’ visit motivation and derivative effects [28]. Previous studies focus only on the influencing factors that affect users’ perception of parks, through which we are difficult to deeply understand the complex process of interaction between park users and the landscape. However, the theory of landscape perception is the classical theory that focuses on the cyclical and interactive process of perception [25], which can help us delve into young adults’ interactive perception of the landscape in urban parks. Therefore, this research constructs a landscape perception model based on landscape perception theory. It combines with human environmental and cognitive processes and the connotative characteristics of spatial perception [29] to provide a theoretical basis for qualitative research on urban park perception, which can help systematically understand young adults’ landscape preferences for urban parks. This model (Figure 1) is divided into three parts: landscape, human, and human–landscape interactions, each of which are explained in detail as follows.

#### 1.3.1. Landscape

“Landscape” is the object of a perceptual process that includes not only the physical environment in urban parks that can be perceived by a human but also crowds who are active in the parks [25]. To some extent, the perceived frequency of various environmental features and crowds can reveal the public’s main concerns in urban parks [8,30]. Furthermore, these concerns imply public preferences [20,31]. Research has found that parks’ environmental features and social factors can influence teenagers’ active and social park use [3]. In particular, the sports features of environmental features and the safety of social factors in urban parks obviously impact the use of parks for physical activities and social interaction [32]. Although research has highlighted specific park characteristics as essential for park use, critical park features may differ among youth sub-groups [33,34]; thus, the main concerns and landscape preferences of young adults may differ among teenagers. Therefore, this part of the model will help research young adults’ concerns and landscape preferences in urban parks and find the critical factors influencing their active and social park use.

#### 1.3.2. Human

Perception is a process of acquiring awareness or understanding perceptual information [35], and environmental psychology divides the human perception process into four steps, namely “sensation–awareness–cognition behavior” [29]. As the origin and basis of complex cognitive activities, the sensory system [36] corresponds to the common senses of sight, hearing, smell, taste, and touch in daily life, all of which are sources of information and sensory modalities in urban parks. Based on sensation, awareness is a comprehensive reflection of everything humans perceive with the help of existing knowledge and experience. Combined with the perceiver’s cultural background, concepts such as site situation, thinking ability, emotional processing, and logical reasoning help transform awareness into cognition [26]. Therefore, cognition is reflected in the complex cognitive activities such as emotion, preference, and memory generated by people in urban parks and is also the focus of this research model.

The public perceptions of urban parks are emotional connections between humans and nature [37]. Both positive and negative emotions can provide a relatively direct and accurate description of public attitudes and preferences towards urban parks, which is essential for informed and responsive landscape decision-making and management [38,39]. Place attachment is the emotional connection that people show linkage to local parks and natural areas [40]. Moreover, it is an important theory for studying people’s emotional connection to places, which stems from emotions or personal upbringing [37]. Therefore, this model also focuses on young adults’ sensations, positive and negative emotions, and the place attachment to urban parks to gain a deeper understanding of young adults’ emotional connections to urban parks.

As an important addition to existing research on environmental characteristics, the above perception steps indicate their subjective perceptions of urban parks, helping to understand young adults’ perceptual preferences and characteristics. As the final step in spatial perception, behavior incorporates into the human–landscape interactions section in landscape perception theory, which is described in more detail in the next section.

#### 1.3.3. Human–Landscape Interactions

Behavior is the action of individuals in response to their perceived environment, depending on factors such as their interests, goals, needs, values, and social norms, reflected in the activities that people generate in urban parks, which are among the tangible states in which the landscape affects people [25]. People’s activities in parks, including physical activities (e.g., running, walking, etc.) and social activities (e.g., chatting, singing, etc.), are beneficial for the young’s physical health and psychological development [3]. On the other hand, such a psychological state of health and wellbeing is the landscape’s influence on people [25]. Our research for young adults’ landscape preferences is for urban parks aiming better to promote young adults’ physical and mental health. Therefore, it is also important to research how urban parks promote young adults’ physical and mental health, which requires understanding how nature improves people’s health. In terms of linking nature exposure to improved health outcomes, prevailing theories on the underlying mechanisms can be summarized as follows [41,42]: (a) provide opportunities for physical activity; (b) the stress reduction theory [43]; (c) attention restoration theory [44]; (d) restorative benefits on mood and cognitive function [45]; (e) the social cohesion theory [46,47]. In this paper, we analyze the health-related perceptions of young adults in urban parks in terms of these five potential mechanisms above and explore the health benefits of urban parks for young adults.

Human–landscape interactions include not only the effects of the landscape on people but also the changes that people cause to the landscape environment, which are generally reflected in the human landscape resulting from the adaptation, use, and transformation of nature during the long-term interaction between people and nature [48]. However, due to the short period and less obvious changes to parks, such an aspect is not the focus of this research for the landscape perception of a group of young people.

### 1.4. Study Focus

Based on the landscape perception model described above, this study qualitatively analyzes the perception reviews of young adults (aged 20–24 years old) in urban parks to understand young adults’ landscape preferences for urban parks and the health benefits they deliver to young users. Specifically, this study answers the following questions:

1. Which landscapes are most popular with young adults, and what are the characteristics of urban parks as perceived by this age group?
2. What health benefits do urban parks bring to young adults?

## 2. Materials and Methods

### 2.1. Study Area

**Figure 2:**
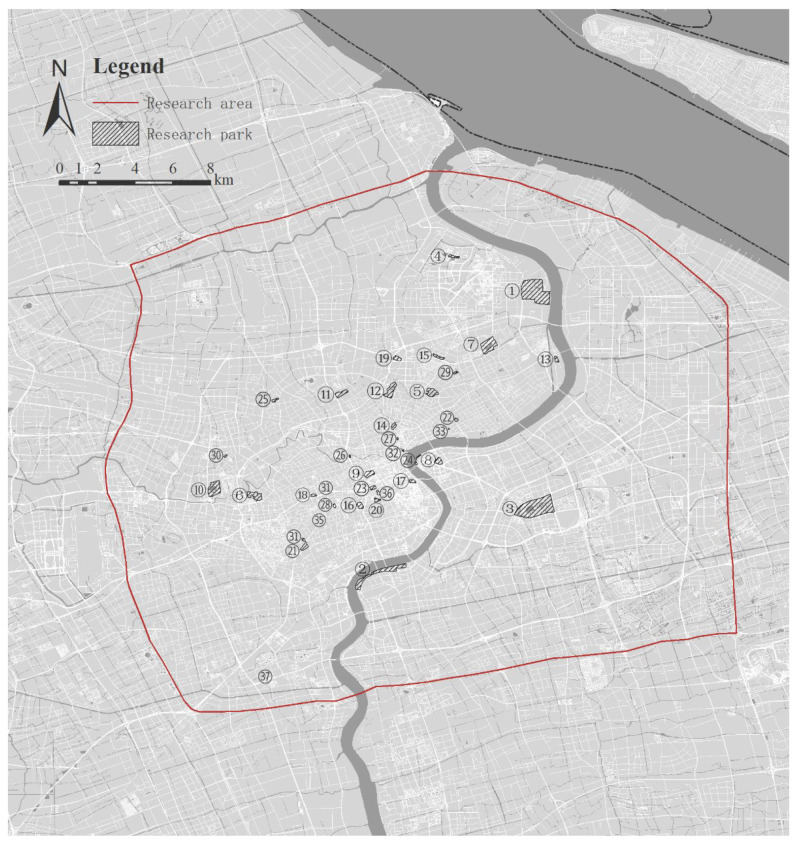
Study areas are urban parks in central Shanghai.

This research was conducted in Shanghai, China, located in the eastern part of China, with an area of 6340.50 km^2^ and a permanent population of 24.28 million. The built-up area of Shanghai has a green coverage of 39.6% and a park area of 8.3 square meters per capita [49]. Shanghai is improving the urban and rural park system by building city parks, regional parks, community parks, and green street spaces in the central city [50]. More than 85% of Shanghai’s urban parks are within 10 ha, mostly distributed in the city center, located within the outer ring road, with an area of 664 square kilometers. Additionally, park visits within the center of Shanghai have increased recently, making it more representative and suitable for research [21]. The urban green space system includes three different scales and classes of parks city parks, regional parks, and community parks in central Shanghai. To ensure the universality and representativeness of the urban park perception research, 37 parks and green spaces of different scales in central Shanghai were selected for this study (Figure 2). The sites range from 0.5 to 100 ha, covering 3 scale ranges and classes, including city parks (12), regional parks (13), and community parks (12) in the central city. As shown in Table 1, the number of selected parks in the three classes is equal, covering different park characteristics, such as children’s parks, sports parks, historical parks, and so on. Participants, who were mainly young college students aged 20–24, were free to choose the parks and were asked to complete the interviews independently after visiting the parks individually or in pairs to prevent the presence of other people from biasing the participants’ responses.

### 2.2. Data Collection

We chose interviews and workshops for data collection, which have been considered effective and important methods in narrative research [51]. They allow participants to communicate their experiences of how they feel about events and environments through speech, text, and audio–visual media. For the interviews and workshops, the ethics review and approval were provided by Tongji University. All the participants have signed the consent form, and all recognizable task features have been specially saved. We have numbered the respondents to avoid the disclosure of identifiable information in the following research.

Three open-ended questions were distributed to participants in the interviews and workshops to guide their perceptions of urban parks. The questions concerned were as follows:

- We explored various sensations in the park by asking participants to record their first feelings upon arrival.
- We learned about perceptions of the landscape in the park by leading participants to keep track of the landscape elements, attractions, people, and activities after taking a tour of the park.
- We collected emotions, feelings, and retrospective reflections evoked in the park by asking what participants liked or disliked in the park in addition to their reflections.

These three questions were designed to guide participants in capturing their own experiences in the park. They were subdivided into many smaller questions under three broad questions (e.g., “What did you see, hear, or smell?” and “What do you find attractive about people, objects, and events?”). Then, participants could record these experiences and perceptions as carefully and comprehensively as possible.

Compared with checklists or structured scales, the use of open-ended questions not only could help capture participants’ own words but also aids in observing participants‘ most authentic perceptions. The study was conducted over several years, with face-to-face questionnaires from September to October of each year from 2014–2020, as participants chose the urban parks to visit by themselves. Participants perceived different spaces in the parks and personally participated in some activities in the parks, responding to the questions asked during and after the visit and recording all answers. The participants completed the interview independently and recorded the answers verbatim. Additionally, we provide them with opportunities to communicate in spoken language and multimedia during workshops. The entire interviews and workshops were recorded from 200 to 1500 words, accompanied by live pictures and audio recordings.

As shown in Table 2, a total of 353 park users participated in this study, with 349 valid interviews. The majority of participants were young college students aged from 20 to 23 years old. In total, 312 participants conducted the study before the outbreaks of the COVID-19 epidemic, while 37 participants conducted it after. Of the 349 participants who took part, 63% were visiting alone, and 26% considered themselves daily users. All 353 participants answered questions about feeling in the park and recording elements and attractions of the park: 65% of the participants answered in detail about the emotions and feelings elicited in the park, 29% mentioned them in the form of a narrative in response to the question about emotions and feelings, 6% did not specify, 84% of the participants answered information such as park review reflections, and 16% of the participants did not answer.

### 2.3. Data Analysis

First of all, we have drawn on the traditionally interpretive approach of thematic analysis [52]. The audio recordings of the interviews were transcribed verbatim, and all the data from interviews and workshops were recorded into Nvivo10 for coding, with all texts coded both inductively and deductively. The researchers created a preliminary coding framework based on the three interview questions mentioned above, and the coding results were integrated into a landscape perception model (Table 3). The framework was iteratively adapted throughout the coding and analysis process as new content emerged. Once the results of the interviews were coded, the researchers adjusted them according to the new categories and subcategories generated frequently [3]. Participants in workshops were also involved in coding photos, drawings, and voice recordings. Various forms of materials were analyzed for meaning using participants’ explanations and our understanding of the context [53].

In addition, data were also analyzed using ‘diffractive’ analysis [54], which has been used in qualitative studies involving interviews with youth age groups [3,9]. This kind of method allows a simple reflection or mirroring of young people’s experiences and perceptions in urban parks. As a result, a researcher’s involvement and analyses, as in wave diffraction, invite original ‘waves’ partly remaining (original material of the words, pictures, and voice recordings from participants). At the same time, these researcher involvements act as obstacles that bring in changed and new ‘wave’ movements (our themes and codes in thematic analysis and our ideas) [9].

We use thematic analysis and statistics on the frequencies of each theme and code (repeated mentions were recorded only once per respondent) and the proportion of them [3,28], followed by using ‘diffractive’ analysis to interpret original materials to comprehend the underlying significance [55], with any disagreements discussed to reach consensus.

## 3. Findings

### 3.1. What Is the Overall Impression of Young People in the Park?

**Figure 3:**
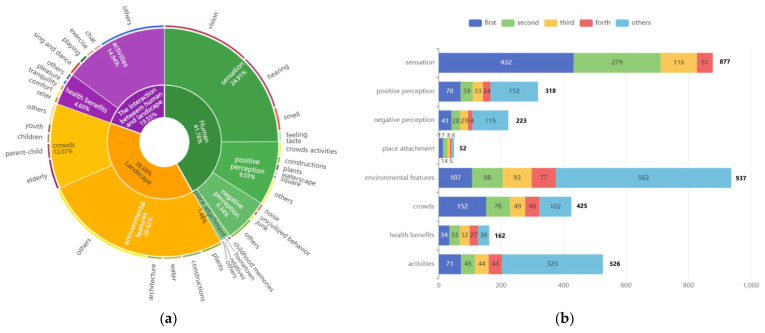
The proportion and frequencies of the young adult’s parks perception. (**a**) The overall proportion of the young adults’ park perception; (**b**) The frequencies of the young adult’s parks perception. The perceived frequencies are ranked from high to low, and the codes of high frequencies can be queried in Table 2, corresponding to this graph.

In our research results, the participants’ subjective perceptions (“human”) accounted for the largest share of codes, accounting for 41.76%. The perceptions of landscape objects (“landscape”) accounted for the second-largest share of codes, reaching 38.69%. The perceptions of “the human–landscape interactions” accounted for the smallest share of codes, accounting for 19.55%. In the breakdown, the participant’s perception of the landscape environment features was the most coded, and the sensory perception (visual, hearing, smell, etc.) was the second most coded. On the other hand, the participants generated the least perceptions about place attachment and health, as shown in Figure 3a.

As shown in Figure 3b, Table 3, we counted the codes in each theme and summarized the top four codes with the highest number. Accordingly, we found that young adults pay more attention to natural landscape elements such as plants and water and various artificial landscape elements. Moreover, young adults’ perception of crowds also accounted for a significant proportion.

Young adults’ sensory in urban parks occupies the largest share of the participants’ subjective perceptions. In general, young adults generated more positive emotions than negative emotions in parks. We summarized several types of landscape elements that made young adults feel positive or negative (Table 4). Additionally, we also found that young adults’ place attachments in urban parks are more likely to be personal memories and emotions.

The codes of crowds’ activities account for the majority of the human–landscape interaction with various types of activities. At the same time, we often see enjoyable feelings such as “relaxation”, “comfort”, “tranquillity”, and “pleasure “ in the interviews. These states are often difficult to perceive, but they are also the health benefits and wellbeing that come from the landscape perception of urban parks.

### 3.2. What Kinds of Landscape Do Young Adults Concerned about?

**Figure 4:**
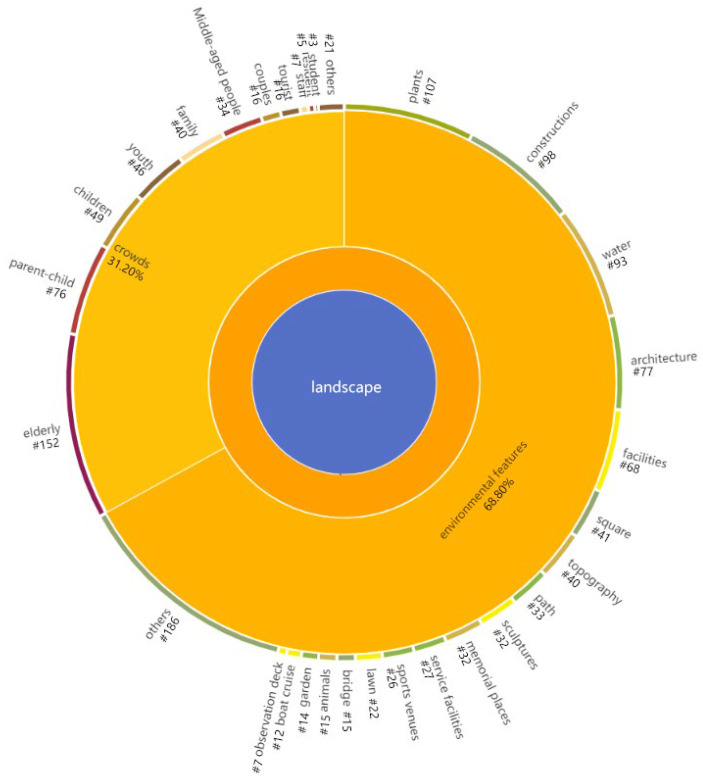
Landscape coding diagram.

It has been widely concerned about the influence of natural attributes and environmental features of urban parks on landscape perception. Existing studies have focused more on the environmental features of parks that attract youth. Our study refined these environmental features and extracted several categories of landscape elements that young adults are more concerned about (Figure 4). Furthermore, we found that young adults pay more attention to natural landscape elements such as plants and water and a variety of artificial landscape elements such as structures, plazas, and facility vignettes. At the same time, the results of our study showed that young adults’ perception of crowds also accounted for a significant proportion. It can be learned that young participants pay more attention to the groups of people with warm relationships, such as the elder, children, and their peers in the park.

#### 3.2.1. Environmental Feature

Table 5 summarizes these landscape environmental features that young adults are most concerned about and the participants’ descriptions of them. Commonly, green plants give respondents feelings of seclusion and openness, while the trees behind respondents give them a sense of shelter. Additionally, many respondents mentioned that seeing an open lake makes them feel very comfortable, for the reason that being near the mountains and water creates a sense of security and warmth. According to the results, young adults pay attention to the natural landscapes, not only because these environmental features give them a spiritual experience of being close to nature but also due to a sense of shelter and security. Meanwhile, there are two main types of movement and stillness in describing the artificial landscape. Some respondents believe that the promenade brings a strong sense of life, “as if time has stood still”. Conversely, for the respondents who love extreme sports, the soul of the park is the place that provides the sports site.

#### 3.2.2. Crowds

The characteristics of young adults’ perception of the crowd are mainly reflected in their attention to the elderly and children, such as their intimate and warm way of getting along. These expressions tie in with personal memory and life experiences [40]. The scene of the elderly and children, which recalls childhood memory, attracts Respondent No. 313 very much:

*A little girl was catching fish with a net under the weeds by the river, while her grandmother stood by and held her hand. “Did you catch this? “ The little girl replied. “Are you going to take them home and fry them up for dinner?” It’s so interesting that a park can participate in the daily life of the residents in such a way.*

(Respondent No. 313)

At the same time, we also heard that many respondents were attracted to others because they brought happiness and joy to the people around them and were moved by his serious attitude toward life. Parks provide young adults the opportunity to connect with others and have social cohesion [46], which makes Respondent No. 316 concerned:

*the person and event that attracted me most was the old grandfather playing the saxophone…because he added another kind of plain and happy atmosphere to the surrounding environment…*

(Respondent No. 316)

### 3.3. How Do Young Adults Perceive the Urban Park?

#### 3.3.1. Sensation

**Figure 5:**
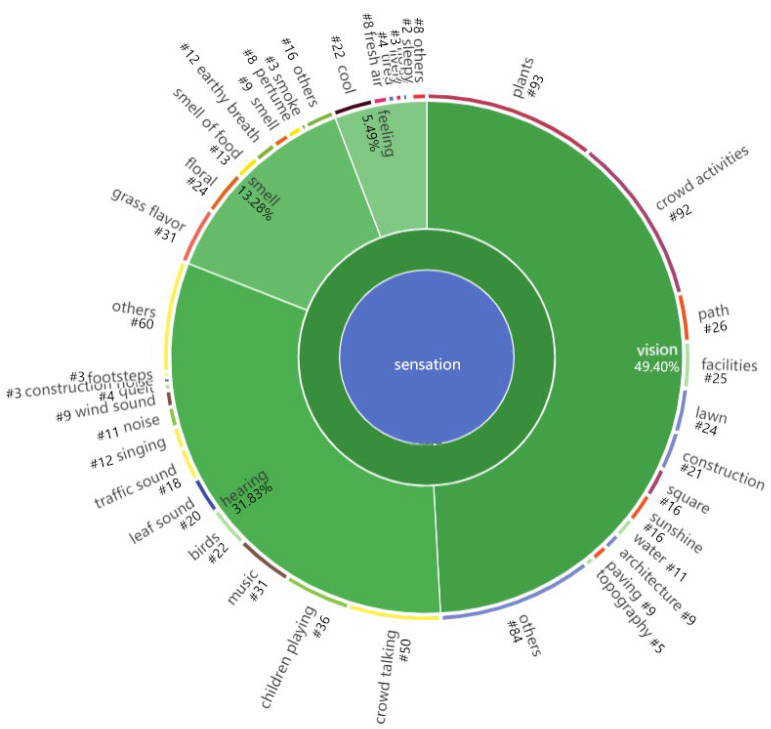
Sensation coding diagram.

The research found that in the sensation by which young adults perceive the external environment, the largest proportion is visual (49.40%), followed by hearing (31.83%), smell again (13.28%), and body feeling at the bottom (5.49%), and there is almost no code about taste. Overall, the proportion of hearing and smell in our research is much higher compared to other research studies, as shown in Figure 5. We also found that young adults’ multiple sensory perceptions are more concerned about the natural landscape, such as the visual codes of plants, the hearing codes of the sound of leaves, and the smell codes of the smell of grass and flowers. Additionally, young adults’ multiple senses are also sensitive to active crowds, such as vision codes for crowd activity, hearing codes for crowd conversations and children playing, and smell codes for crowd smell and perfumes.

Among the multiple sensory perceptions, young adults are more sensitive to hearing and smell in urban parks. Sound is very important to landscape perception for people such as Respondent No. 309. The sound enhances their perception of environmental features in urban parks. Their expression makes it clear that the physical characteristics of visual landscapes could also have more effect on soundscape perception [56].

*…a gust of wind rustled the leaves of the hanging spruce trees, and the leaves fell off one by one… Even the rustling of the leaves and the crowd chatter could not drown out the sound of birds singing in the park. I noticed the bird sounds when I entered the park but couldn’t find a single bird anywhere.*

(Respondent No. 309)

Likewise, smell greatly influenced participants’ perception of the landscape, which is helpful for defining place [57]. The nice scent brings a transcendent sensory experience to Respondent No. 10, while the nasty smell diminishes Respondent No. 152’s experience of being close to nature. These smellscapes are of concern to the youth, especially are perceived more by the female.

*I can smell the smell of autumn, and the fallen leaves are baked by the sun with a golden rhythm […] There is a vague aroma of cinnamon.*

(Respondent No. 10)

*…and of course, there is a smell that disrupts this beauty, the smell of smoke! I am most disgusted by the smell of smoke in public places, … and this burst of smoke, which yanks me out of nature again, is suffocating.*

(Respondent No. 152)

Additionally, many respondents also want to use their body’s sense of touch to perceive urban parks because it makes them feel nature and culture. Although touch has been traditionally considered unimportant, Respondent No. 318 expresses a strong desire to directly contact nature with both hands, forming a strong memory of the place [58].

… *I kept touching the lake, soil, structures, leaves, flowers, tiles, promenades, etc., with my hands, which made me feel the texture of nature and humanity at the same time.*

(Respondent No. 318)

#### 3.3.2. Emotion

Participants in the interviews expressed their positive and negative opinions about some environmental features and others’ behavior. It can be seen that young adults generated more positive emotions (58.78%) than negative emotions (41.22%) in urban parks.

**Figure 6:**
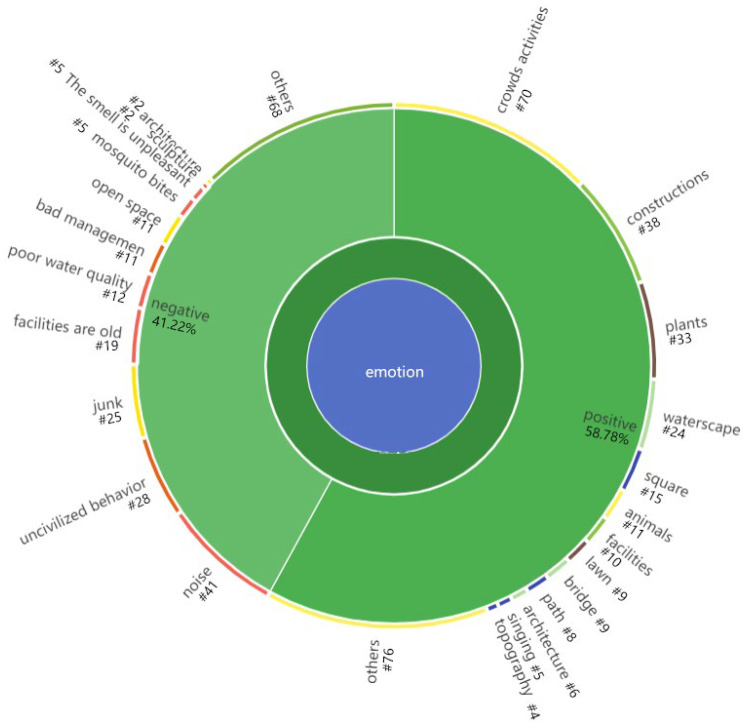
Emotion coding diagram.

As shown in Figure 6 and Table 6, more positive emotions are generated from crowd activities, natural landscapes of water features and plants, and artificial landscapes of squares and structures. For Respondent No. 329, the harmonious picture of a family can make her “very happy”. Respondent No. 339 told us that seeing different people doing their own thing in parks helps her “catch the sense of life “. These elaborations can be seen as the social connections formed from nature, which are characterized by the entanglement of natural experiences with people, especially family and loved ones [9]. The observation of others in the park evokes ‘atmospheres of sociality’ and ’atmospheres of safety and belonging’ [59] among young adults. For these reasons, young adults also like artificial landscapes such as structures and squares because they are places where a lot of activity occurs.

*Large and small squares are the main places where people move around, where many interesting actions occur… and where people interact with each other.*

(Respondent No. 332)

These activities take place in places that attract young adults’ attention and make them think of their loved ones or childhood memories, which can also be explained by the theory of place attachment [40].

*The most attractive object to me is the pavilion on the hill where grandparents play chess and chat in the pavilion feeling very atmospheric and happy… It makes me think of my grandparents, and I hope they can also spend more time with their old friends.*

(Respondent No. 214)

Not surprisingly, the natural landscape is getting a lot of attention from young adults, and many respondents also strongly prefer it. Many respondents were impressed by the trees along the riverside of the park, feeling “wonderful and intimate”. Most respondents were attracted by the combination of the lake with the buildings and plants was very “natural and harmony”. Even some of them considered the park’s water system as the link between the whole park and “connects all the scenes”. From these expressions, we can see that the preference for natural landscapes is applicable to the vast majority of people, and young adults are no exception [17,19,21].

The factors that cause the most negative perceptions among young adults are uncivilized behavior, unhygienic behavior, and loud noise. As we can see from the result, what triggers negative emotions among young adults is not the environmental feature of the park but the behavior of others. We heard respondents believe that he “dislikes some behavior, but this does not originate from the space itself“. From this point of view, it is often not the environment itself that causes negative emotions among young adults in the park but the bad behavior hidden behind it [21]. Many respondents mentioned that the factors of negative emotions also come from various uncivilized behaviors:

*I disliked a variety of uncivilized behaviors, such as a woman lying on a bench and sleeping; many uncles smoking and spitting in the square.*

(Respondent No. 309)

*The event I do not like is still littering anywhere… and I felt that the tourists who made this garbage had no sense of public morality.*

(Respondent No. 338)

*Some visitors always ignore the forbidden sign, so I am worried about this kind of behavior of disregarding safety.*

(Respondent No. 140)

Respondents were also very sensitive to the sanitary conditions of the parks. The dirty and unhygienic environment was also a frequent negative topic, and many of these dirty and unhygienic environments that made them feel uncomfortable came from the uncivilized behaviors mentioned above.

*I do not like some of the park’s functional facilities, such as the garbage collection office, some dirty and untidy, and a bit of a fatalistic scenery.*

(Respondent No. 195)

*The seat under the tree is sticky and can’t be sat on.*

(Respondent No. 311)

Noise is also an important factor that triggers negative emotions among young adults in parks. Our participants mentioned a variety of noises, and the most mentioned were unpleasant emotions about children’s cries, the sound of facilities, and noisy music. Respondent No. 334 even developed an aversion to the whole children’s amusement park because she did not like the noise of the rides. These interviews indicate that the perception of negative emotions caused by noise annoyance can affect the young adults’ overall experience in the park [60].

*I don’t like that a playground borders the park as it’s too noisy.*

(Respondent No. 316)

*I was bothered by the children who scream and make noise on the lawn.*

(Respondent No. 335)

*… the music played by the audio successively were “lousy” songs that could be heard everywhere on the street, so it gave me a noisy feeling, which I did not like.*

(Respondent No. 321)

### 3.3.3. Place Attachment

**Figure 7:**
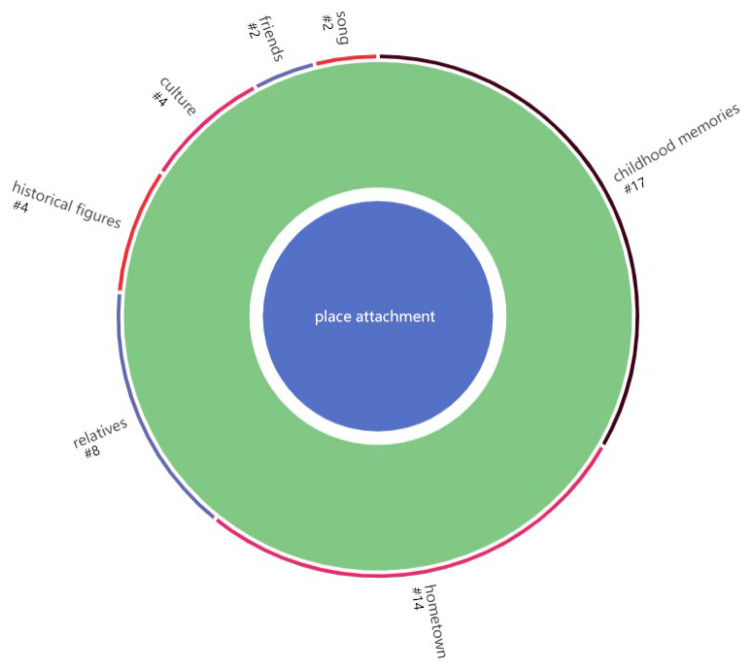
Place attachment coding diagram.

As shown in Figure 7 and Table 7, place attachment generated by young adults in urban parks is more often expressed as place identification with memories and emotions such as hometown, childhood, and relatives, while place dependence can be rarely found [52]. According to the findings of the previous sections, these feelings are also often closely related to the people they are concerned with and the resulting positive emotions [40]. In more cases, it is a mixture of the recall of personal memory and life experiences (e.g., hometown, childhood, relatives), thus establishing an emotional connection with the place [37].

## 3.4. What Kinds of Health and Wellbeing Benefits for Young Adults?

**Figure 8:**
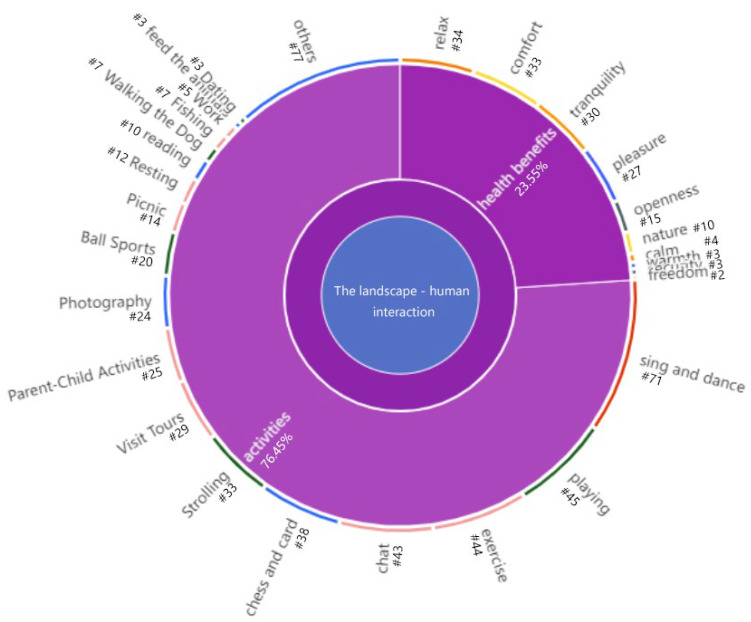
The landscape–human interaction coding diagram.

The impact of parks on young adults is mainly reflected in the behavioral activities in urban parks, the most attractive among which are singing and dancing, exercising, chatting, playing, and so on. (Figure 8). This part of the code accounts for the majority (76.45%) of “the human–landscape interaction”, indicating that physical activity and social interaction in the park are significantly linked with young adults’ health [61]. Additionally, other potential mechanisms exist for people to improve their health through natural contact in urban parks [42]. For example, we often see “relaxation”, “comfort”, “tranquility”, and “pleasure”, in the interviews with young adults. Therefore, it can be seen as intangible impacts of parks, based on which we can describe the perceived health and wellbeing in urban parks.

These diverse descriptions of health benefits generated in our research also provide new insights into landscape perception. We categorized intangible health benefits into four categories (Table 8) based on the potential mechanisms by which human contact with nature improves health: stress relief, attention restoration, restorative perception, and social cohesion [42].

In addition, we also found a significant increase in participants who mentioned crowd activity after the COVID-19 epidemic, along with a significant increase in unhygienic or dirty of their negative emotions. Many respondents’ expressions reveal an obvious improvement in sensitivity to crowd activity and environmental hygiene in urban parks:

*Many people in the square randomly smoked, and spitting.*

(Respondent No. 309)

*The incident that I don’t like is still littering anywhere. A large bag of garbage was thrown under the bench on the roadside, which was very conspicuous.*

(Respondent No. 338)

At the same time, the level of respondents’ perception of the plant landscape in the park after the epidemic was significantly higher compared to before. For Respondent No. 321, it is of significant importance to be surrounded by green peace and tranquility to overcome the difficulties of the epidemic:

*The edge of trees is an extension of the hill… the sunset on such a vast lawn can still give people a sense of beauty, curing my anxiety under the epidemic.*

(Respondent No. 321)

*A small lotus pond by the square, the lotus pond is a symbol of tranquility and beauty, which is extremely precious after the epidemic.*

(Respondent No. 321)

## 4. Discussion

### 4.1. The Application of the Landscape Perception Model Can Better Reveal the Landscape Preferences of Young Adults

We found that the landscape perception model can help study and reveal the public’s perception of urban parks. Current research on young people’s landscape perception tends to focus on the features and physical attributes of the landscape environment that influence park perception [3,13,16] while neglecting how they perceive urban parks. The landscape perception model applied in this study overcomes this limitation. From 2014 to 2020, our interviews with people were not limited to their concerned park environment but also included how they perceived the features. Moreover, their views on urban parks were taken into consideration, including sensory sensations and subjective feelings, such as positive and negative emotions generated. We also focus on the activities and health benefits of human interaction with the landscape. The relevant perceptual results in the interviews are encoded into subdivisions of the model, which can help reveal landscape preferences more intuitively than the perceptual frequencies of environmental features. Therefore, we can better understand young adults’ landscape perception and landscape preferences in urban parks by applying the landscape perception model. Additionally, this repeatable and transferable landscape perception model can be applied to other perception-related studies.

### 4.2. Young Adults’ Landscape Perceptions Are Richer in Diversity and Express More Subjective Feelings

Overall, we found that the percentage of codes reflecting people’s subjective feelings (41.48%) is higher than that of codes describing environmental features (37.16%). The finding indicates that young adults’ landscape perception is not limited to the physical environment, and the subjective feelings caused by environmental features should also be concerned. We also expand the definition of the landscape environment by considering the people that young adults are concerned with. Based on this, we learned that young adults care for vulnerable groups such as the elderly and children and express more concern for the intimate and warm scenes between these groups.

According to psychological studies, the proportion of external information received by the human brain daily through the five senses is 83% for vision, 11% for hearing, 3.5% for smell, and 1% for taste [62]. Our research results show that the ranking of external information perceived in the park is generally similar to the existing psychological studies on the five senses. In contrast, the proportion of hearing and smell is much higher, suggesting that the influence of auditory and olfactory senses on young adults’ perceptions is more prominent in urban parks than in other scenes. Since the capability of multiple sensations decreases with age, we find young adults’ concerns about sound, smell, and touch are more pronounced, especially for females [57]. Therefore, to attract more young people to visit and use urban parks, more attention should be paid to creating sound and smell landscapes in parks. Therefore, future research should also focus on the influence of soundscape and smellscape on young people’s landscape perception [58].

On the other hand, we found the factors that caused the most negative perceptions among young adults in urban parks were uncivilized behavior, unhygienic, and noise. These factors often have little to do with the inherent environmental features of the park but come from the unpleasant crowd activities of others. Young adults are also very sensitive to noise in urban parks, which again confirms the prominent role of hearing in their perceptions of urban parks. As we can see, since the perception of negative emotions caused by noise annoyance can obviously affect the young adults’ overall experience in the park [60], the focus on reducing noise annoyance in the parks should be emphasized.

Place attachment in our research was often expressed as place identification [63] with memories and emotions of hometown, childhood, and relatives, while place dependence was rarely identified. In most cases, such factors have emotional connections with places as they recall their memories and life experiences, which are often closely related to the people they are concerned with and the resulting positive emotions [40]. Furthermore, such emotional bonds act as the intersection of users’ personal experiences with places [37], while there is less identification with and a sense of belonging to local culture.

### 4.3. Young Adults’ Landscape Preferences Are also Related to Behavioral Activities in Addition to Environmental Features

Our research refines the results of existing studies, finding that the landscape elements of urban parks that attracted more interest from young adults are plants, water features, structures, and facilities vignettes. This finding coincides with the findings of existing studies [3,19]. We also found that young adults pay attention to environmental features similar to the public [16,17]. However, our interview further explains the landscapes that young adults prefer in various parks. According to the analysis, young adults focus on plants and water features largely because these natural landscapes provide them with a sense of security and shelter, which can be interpreted as a sense of escape from the city and being close to nature [9].

On the other hand, the preference for artificial landscape is reflected in two types—movements and stillness. The movement is driven by the facilities to provide young adults with a venue for activities (such as recreation and sports), which shows that sports features are environmental characteristics that attract teenagers [3] and have significant impacts on young adults. The stillness is due to the history and cultural perception to bring them a rich spiritual experience [64], which shows the characteristics of young adults differ from teenagers.

In the research, we focused on the positive emotions of young adults in urban parks. Our findings show that crowd activities make young adults develop the most positive emotions. Current studies tend to focus only on the impact of the environmental features [21] but ignore that crowd activities can significantly impact their positive emotions. In many cases, young adults’ attention to other people and activities evokes their own experiences and memories, especially family and loved ones [9]. Additionally, the ‘atmospheres of sociality’ and ’atmospheres of safety and belonging’ brought from others make sense [65] for them as well. Since positive emotion can positively affect a person’s mental health, the findings of this study can support that environments with higher activity levels are more conducive to improving young adults’ mental health to some extent [18].

### 4.4. Urban Parks Are Important for the Physical and Mental Health of Young Adults

Young adults’ physical and mental health will be promoted by participating in sports or social activities in urban parks, which is the tangible part of the interaction between humans and the landscape and accounts for the majority of the human–landscape interaction. It is probably why the existing studies are more deeply explored, and the mechanisms of health benefits are better constructed in physical activities [41,42]. Additionally, other potential mechanisms for improving health through natural exposure in urban parks exist, which are often difficult to detect. We summarized the intangible health benefits perceived by young adults in four categories: stress reduction, attention restoration, restorative perception, and social cohesion. As a result, we found that the most frequent descriptions of relaxation and stress relief take people away from stressful states to generate health benefits [9]. Many participants also mentioned feeling comfortable and peaceful in the park to restore their energy from fatigue. It also can be seen that young adults are more likely to develop place attachment in a preferred natural environment as more descriptions of restorative perceptions appear [37]. Social interaction was less described in our study, but some participants also reported being able to relieve fatigue and rejuvenate after interacting with others and participating in activities.

Research shows a significant increase in the coding number that participants mentioned crowd activity and unhygienic topics after the outbreak of the COVID-19 epidemic. It suggested that participants’ sensitivity to crowd activities and environmental hygiene in urban parks after the epidemic was significantly elevated, mainly due to a general increase in individuals’ risk perceptions after COVID-19 [66]. At the same time, the level of participants’ perceptions of botanical landscapes in parks after the epidemic significantly increased compared to the pre-epidemic period. Existing studies on the health benefits of green spaces proved green spaces have positive effects on people’s mental health [67], and green health efficacy studies have found that botanical landscapes are an important factor in the impact of green space environments on people’s mental health [65]. Therefore, to some extent, the findings of this study reflect that long-term home isolation makes the need for green landscapes that can relieve residents’ anxiety increasingly urgent in the context of the epidemic [68].

## 5. Conclusions

Understanding how young adults perceive urban parks and their health benefits is a prerequisite for better design, planning, and management of urban parks to make them more inclusive. In this paper, we expand the understanding of youth’s perceptions of parks from existing research and find that young adults’ perceptions of parks have differences from teenagers. Based on our research, we suggest that park planners and policymakers should consider young adults’ perceptions and propose measures (Table 9) to improve the perceived experience of young adults in urban parks from three aspects. It is helpful to create beautiful water features, plants, and other natural landscapes and promote parks’ ability to accommodate activities, especially more dynamic sports and recreational facilities. In addition, improving park management should control uncivilized behavior, unhygienic problems, and noise in parks, all of which will seriously affect the perception and use of parks by young adults. It is also necessary to pay more attention to creating sound and smell landscapes in urban parks, which we have found to be more attractive to young adults in urban parks. More young adults may be attracted to parks by adapting to young adults’ perceptions, which could benefit their physical and mental health and alleviate their health problems to some extent.

Additionally, this paper applies a landscape perception model to comprehensively understand urban parks’ landscape perception. It overcomes the limitations of previous studies that focused only on landscape environmental features and expands the application of landscape perception theory to evaluating urban park use, reflecting the complexity of landscape perception. In the future, this reusable landscape perception model can be applied to other perception-related studies to understand people’s perceptions of urban parks better.

There are some limitations in this study: (1) the sample size of this study is limited because the traditional interview questionnaire obtained fewer data. On the other hand, the participants are mostly university students, which is limited to representing the young people group to a certain extent; (2) the research results are still somewhat generalized. This study does not consider the differences in various park characteristics, such as historical and sports parks’ specificities.

This study lays the foundation for quantitative research, and the landscape perception model can be applied in the future to conduct in-depth quantitative research on the impact mechanisms between landscape perception and health benefits. We also encourage the integration of web data with traditional interview results to obtain more convincing results. In addition, future studies can consider the public perception of different types of urban parks, which would allow for more refined research results.

## Figures and Tables

**Table 1:** The Urban parks information table.

Class	Serial Number	Park Name	Area	Park Features
City park	1	Gongqing Forest Park	131 ha	National forest park
	2	Expo Park	200 ha	Expo culture
	3	Century Park	140.3 ha	
	4	SMP Skate Park	14.7 ha	Sports park
	5	Peace Park	17.6 ha	
	6	Zhongshan Park	20 ha	
	7	Huangxing park	40 ha	
	8	Lujiazui Central Green Space	10 ha	
	9	People’s Park	35.1 ha	
	10	Changfeng Park	36.4 ha	
	11	Zhabei Park	13.7 ha	
	12	Lu Xun Park	28.6 ha	History and cultural park
Regional park	13	Fuxing Island Park	4.2 ha	
	14	North Sichuan Road Park	4.2 ha	
	15	Siping Science and Technology Park	7.0 ha	
	16	Fuxing Park	8.9 ha	History and cultural park
	17	Ancient City Park	4.0 ha	History and cultural park
	18	Jing’an Park	3.4 ha	Sculpture
	19	Quyang Park	6.5 ha	
	20	Taipingqiao Park	4.4 ha	
	21	Xujiahui Park	8.5 ha	History and cultural park
	22	National Anthem Memorial Square	2.7 ha	
	23	Yanzhong Green Space	3.5 ha	
	24	Pudong Riverside Park	8.0 ha	Riverside park
	25	Ganquan Park	3.2 ha	
Community park	26	Jiuzi Park	0.5 ha	Cultural and educational park
	27	Kunshan Park	0.3 ha	
	28	Xiangyang Park	2.2 ha	
	29	Pinehurst Park	1.4 ha	
	30	Lancashire Park	1.3 ha	Youth culture
	31	Hengshan Park	1.2 ha	
	32	Chinese Park	0.4 ha	
	33	Huoshan Park	0.4 ha	Cultural and educational park
	34	Suzhou Road Children’s Park	0.5 ha	Children’s park
	35	Urumqi Road Children’s Park	0.5 ha	Children’s park
	36	Huaihai Park	1.5 ha	Cultural and educational park
	37	Xuhui Jiachuan Road Garden	0.5 ha	

**Table 2:** Respondents’ demographic table.

Respondent Characteristics	N (Total *n* = 349)	Percent
Sex		
Male	94	26.9%
Female	255	73.1%
Age		
19	13	3.7%
20	169	48.4%
21	98	28.1%
22	62	17.8%
23	6	1.7%
24	1	0.3%
Country of birth		
China	347	99.4%
others	2	0.6%
Participation year		
2014	34	9.7%
2015	54	15.5%
2016	44	12.6%
2017	45	12.9%
2018	68	19.5%
2019	48	13.8%
2020	56	16.0%
How to visit		
Alone	220	63.0%
With partner	129	37.0%
Daily park user		
Y	91	26.1%
N	258	73.9%

**Table 3:** Encoded classification table of interview.

Dimension	Classification		
Landscape	Recognizable attractions and elements in the park	Recognizable crowds in the park	
Human	What you saw, heard, smelled, and felt	What you like and dislike	Elements relate to developing the place attachment
The human–landscape interaction	The activities and behaviors you take part in	The description of health and wellbeing	

**Table 4:** Young adults’ perception encoding table. The code number and proportion of the young adult’s parks perception for each dimension\theme\code, “#” stand for code number, ”%” stand for proportion, we chose the 4 codes with the most frequency for each theme to represent, and the rest were put into “others”.

Dimension	#	%	Theme	#	%	Rank	Code	#
Human	1470	41.76%	Sensation	877	24.91%	First	Vision	432
						Second	Hearing	279
						Third	Smell	116
						Forth	Feeling	51
						Others	Taste	0
			Positive perception	318	9.03%	First	Crowd activities	70
						Second	Constructions	39
						Third	Plants	33
						Forth	Waterscape	24
						Others	Others	152
			Negative perception	223	6.34%	First	Noise	41
						Second	Uncivilized behavior	28
						Third	Junk	25
						Forth	Bad management	14
						Others	Others	115
			Place attachment	52	1.48%	First	Childhood	17
						Second	Hometown	14
						Third	Relatives	8
						Forth	Historical figures	5
						Others	Others	8
Landscape	1362	38.69%	Environmental features	937	26.62%	First	Plants	107
						Second	Constructions	98
						Third	Water	93
						Forth	Architecture	77
						Others	Others	562
			Crowds	425	12.07%	First	Elderly	152
						Second	Parent–child	76
						Third	Children	49
						Forth	Youth	46
						Others	Others	102
The human—landscape interaction	688	19.55%	Health benefits	162	4.60%	First	Relax	34
						Second	Comfort	33
						Third	Tranquility	32
						Forth	Pleasure	27
						Others	Others	36
			Activities	526	14.94%	First	Sing and dance	71
						Second	Playing	45
						Third	Exercise	44
						Forth	Chat	43
						Others	Others	323
							Total	3520

**Table 5:** Most common descriptions of landscape environment features.

Trees and Plants	Water	Artificial Landscape
The greenery gave respondents two completely different experiences: quiet and open. *“compared to the quiet and far-reaching green space … this green space is more open …”* (Respondent No. 321)	Respondents believe that water is aesthetically pleasing *“ water is the most praiseworthy for this park.…as if a few more steps and we are at the water’s edge again.”* (Respondent No. 315)	Structures make for a rich spiritual experience *“There is a small house … reflects the meaning of “a great hermit in the city …”* (Respondent No. 319)
The greenery at the lake gives respondents a sense of security. *“the taller trees behind the chairs … give people a sense of shelter.”* (Respondent No. 94)	Water features make park users feel tranquil and comfortable *“There are many waterside landscapes … which makes people feel a classical mood.”* (Respondent No. 331)	Seats, scenic walls, and other facilities also have a strong appeal *“What attracted my attention is that the square’s scenery is curved…”* (Respondent No. 309)
Tree roots left a deep impression on respondents *“There is also a deep memory of the riverside under the trees… feeling the ups and downs brought about by the interlocking roots, wonderful and intimate.”* (Respondent No. 336)	Wang Yulu likes the lake and mentions the security of the mountains *“I like the lake and the many trees next to it… a good place is “by the mountains and water” is true.”* (Respondent No. 339)	For Yaoqi Zhang, who loves skateboarding, the buildings and structures that provide the venue are necessary *“… I think the soul of the skatepark is the structures that provide the skatepark”* (Respondent No. 8)
Plants also provide rich colors *“Many flowering shrubs are planted along the riverbank, which also adds diverse colors along the way.”* (Respondent No. 214)	The open lake makes people feel comfortable *“I am very pleased when facing the open lake, with the beauty of the view…”* (Respondent No. 195)	The park’s amusement facilities are also popular with youth *“… I was still attracted by the top of the rotating swings in the playground.”* (Respondent No. 339)

**Table 6:** Young adults’ favorite landscape types and descriptions.

Crowd Activities	Natural Landscape	Artificial Landscape
The image of family members together in harmony and warmth is also very attractive to the participants *“A family of three, sitting on a boat… the child smiled, the family smiled, this scene is very harmonious, very happy. “* (Respondent No. 329)	The water features surprised participants, *“…The spatial experience of the whole environment is very surprising, and the architecture and plants match naturally and harmoniously.”* (Respondent No. 1)	Many participants mentioned liking the view and activity attributes of the square *”Spring Square is my favorite landscape area”* (Respondent No. 43)
Respondents believe that seeing different people and activities is the main reason they like urban parks *“I always feel that seeing these different attributes of people gathered here, walking, chatting, doing their own little things, is something very firework. Thus, I like parks in the city.”* (Respondent No. 339)	Many participants clearly expressed their love for various water features. *“One of the major features of the garden is its water, and the whole garden is tied up with water to string together various views.”* (Respondent No. 224)	Respondents love the pavilion and the elderly there and think of their own relatives as a result. *“The thing that attracts me most is the pavilion on the hill…the elderly need a space or a place to meet old friends, and this pavilion is a good medium. It reminds me of my own grandparents.”* (Respondent No. 316)

**Table 7:** Young adults’ place attachment to urban parks.

Hometown	Childhood Memory	Relatives
The leisurely activities in the park caused respondents to cherish their hometown *“… I was reminded of my hometown, a frontier town in a third-tier city, whose peace and calmness is cherished.”* (Respondent No. 315)	The park made respondents seem to go back to his childhood memories *“When I sat down…my first thought was as if I had gone back to the park I used to go to in my childhood”* (Respondent No. 321)	Respondents will be happy when he thinks of their relatives in a park *“If there is a park like this near my home, my grandparents must be very happy. They can dance, play chess and chat with their old friends every day”* (Respondent No. 316)
The rich atmosphere of the park makes respondents, who are studying abroad, feel like he has returned to their hometown. *“I felt a very strong sense of life, which made me feel as if I was back in my childhood hometown”* (Respondent No. 329)	The current park scene reminds respondents of the space where he first played as a child. *“the place where I first played as a child was like the lawn… It’s a pity that I don’t have the same heart and partners.”* (Respondent No. 326)	The opera heard in the park made Respondents recall their grandfather *“Listening to the old man play the erhu and sing the Qin cadence…I recalled the sound of her grandfather…”* (Respondent No. 99)

**Table 8:** Young adults’ place attachment to urban parks.

Stress Relief	Attention Restoration	Restorative Perception	Social Cohesion
The park makes respondents feel relaxed physically and mentally. *“… there is a sense of ease while leaving the noise. The lake was calm, … and the body and brain were very relaxed.”* (Respondent No. 315)	The park allows respondents to experience a quiet atmosphere. *“…I felt as if the outside world’s hustle and bustle was isolated, I can feel only the mysterious quiet atmosphere with birdsong.”*(Respondent No. 321)	Respondents saw that her favorite kites caused childhood memories and happiness. *“…kites have always been associated with happy times in childhood…I looked at them to re-experience the happiness as a child. “* (Respondent No. 240)	Respondents feel happy and at ease when being part of the activities in this field. *“…when I am part of the activities on this field …it is lively and the happy atmosphere comes over me and makes me feel at ease in this field.”* (Respondent No. 316)
Stress can be relieved by looking into the park. *“Looking into the distance is very relaxing for both body and mind, and it is not what you see but the scene you see.”* (Respondent No. 318)	Anxiety and tiredness were restored in the park. *“This park makes people feel at ease, and my restless and tired mind can be relieved here.”*(Respondent No. 10)	Respondents’ minds gained peace in their favorite environment. *“… quietly feeling the wetness of the grass…. At that time, my heart gained a lot of peace.”* (Respondent No. 138)	After interacting with the children, many respondents were very happy. *“Feeding the pigeons and interacting with the children was very happy and joyful.”* (Respondent No. 94)
*“highly tense nerves finally relaxed at the moment.”* (Respondent No. 10)	*“The whole atmosphere is peaceful and calm, with happiness and quietness of years.”* (Respondent No. 332)	*“ My memories of such places are always sweet and fragrant… remind me of the amusement parks of my early childhood.”* (Respondent No. 339)	Talking with friends can be very relaxing. *“It’s very relaxing to talk with friends, naturally and eloquently.”* (Respondent No. 310)

**Table 9:** Measures to improve the perceived experience of young adults in urban parks.

Theme	Finding	Measures	Category
Environmental feature/emotion	Prefer nature	Create beautiful water features, plants, and other natural landscapes.	Landscape environment creation
Sensation	Richer in diversity	Pay more attention to creating soundscape and smellscape in parks.	
Health benefit	Dynamic and static are equally account	Offer the possibility of social interaction and solitude at the same time.	
Activity/emotion	Concern for behavioral activity	Promote parks’ ability to accommodate activities.	Park activities planning
Environmental feature/activity	Prefer dynamic and exercise	Provide more dynamic sports and recreational facilities for young adults’ demand.	
Crowds/place attachment	Concern for memories of childhood	Provide more family or parent-child activities to resonate with young adults easily.	
Emotion/activity	Dislike bad behavior	Make more initiatives to control uncivilized behavior.	Park management
Emotion	Focus on sanitation issues	Deal with unhygienic problems to keep the environment tidy.	
Emotion/sensation	Sensitive to noise	Reduce noise annoyance through good layout and noise management.	

## Data Availability

The data that support the findings of this study are collected from the participants involving personal privacy, which were used under license for the current study, and so are not publicly available.
